# Antiobesity and Anti-Inflammatory Effects of Orally Administered Bonito Extracts on Mice Fed a High-Fat Diet

**DOI:** 10.1155/2017/9187167

**Published:** 2017-10-24

**Authors:** Emi Ikebe, Nichole Fife-Koshinomi, Takashi Matsumoto, Takaaki Yahiro, Taichi Ikebe, Hidekatsu Iha

**Affiliations:** Department of Microbiology, Faculty of Medicine, Oita University, Yufu, Oita 879-5593, Japan

## Abstract

**Background:**

The condensed fermentative extract of bonito (BoE), skipjack tuna* (Katsuwonus pelamis)*, has claimed its health conditioning effects against lifestyle-related diseases such as hypertension and type 2 diabetes.

**Methods:**

We evaluated the antiobesity and anti-inflammatory effects of BoE on mice fed a high-fat diet (HFD). Mice (9 weeks of age) were maintained for 11 weeks on HFD with or without BoE (50 mg or 500 mg/kg).

**Results:**

Compared with untreated mice, BoE50 or BoE500 mice achieved maximum weight reductions of 7.4% (males) and 11.4% (females), and visceral fat in male BoE500 mice was more decreased among all mice (*P* = 0.00459). Furthermore, an antiobesity gene uncoupling protein-1 was significantly induced in the visceral fat tissues of male BoE500 (*P* = 0.0110) and female BoE50 and BoE500 mice (*P* = 0.0110 and *P* = 0.0110, resp.). Finally, we detected reduced amount of granulocyte-colony stimulating factor (*P* = 0.0250) in the sera of female BoE50 and interleukin- (IL-) 5 (*P* = 0.0120), IL-6 (*P* = 0.0118), and IL-13 (*P* = 0.0243) in female BoE500 mice.

**Conclusion:**

The antiobesity and anti-inflammatory effects of BoE were demonstrated with our examination system and any toxic adverse effects were not observed in mice during the 3-month investigation.

## 1. Background

Fermented dried bonito and its hot water extracts (referred to as BoE hereafter) have been one of the most traditional and popular condiments and BoE in folk remedies has served as a nourishing tonic for years in Japan [1]. The accumulating evidences of BoE's health-promoting effects have been demonstrated in preclinical models such as preventative effects on ovarian hormone deficiency-induced hypercholesterolemia [2], alleviation of atopic dermatitis-like skin lesions [3], and amelioration of type 2 diabetes mellitus-induced bone frailty [4] and clinically for significant reduction of hypertensive volunteer's blood pressures [5]. Because of its fermentation process, BoE consists of condensed amino acids and peptides (60%), ashes (6%), lipids (4%, mostly polyunsaturated docosahexaenoic acid (DHA), and eicosapentaenoic acid (EPA)) [4]. DHA and EPA exert anti-inflammatory, antiobesity, and protective effects on patients with metabolic syndrome or cardiovascular and cerebrovascular diseases [6, 7].

The manufacturing of dried bonitos requires multiple boiling steps, fermentation and washing procedures, and yields condensed BoE. A powdered form of condensed BoE with reinforced dried bonito powders has been manufactured as a dietary supplement. While BoE contains high amount of amino acids and peptides including anserine [8] and other unidentified components (Supplemental Table 1 in Supplementary Material available online at https://doi.org/10.1155/2017/9187167), it also contains relatively high amount of sodium chloride and arsenic which is associated with multiple adverse effects on our health such as neurotoxicity and carcinogenicity [9, 10]. Mutually exclusive features of BoE for health based on the previous reports are still to be solved and that prompted us to evaluate the antiobesity/anti-inflammatory function (health promoting) or effects on liver function (noxious) of BoE with an experimental animal model.

We fed mice with high-fat diet (HFD) to induce obesity and treated BoE for three months. The effects of BoE supplementation were evaluated with following six criteria: (1) body weight, (2) dietary bulk, (3) body fat (visceral and subcutaneous), (4) biochemical values, (5) the levels of cytokines and chemokines in sera, and (6) the uncoupling protein-1 (UCP-1) gene [11] expression profiles of lower abdominal adipose tissues. BoE treated group showed statistically significant facilitation of food consumption; however increased body weight or body fat was not detected. BoE treated group did not show any deteriorating biochemical signs in sera or obesity symptoms but rather downregulating the production of several inflammatory cytokines. BoE also showed ameliorating visceral fat amount and increased UCP-1 expression although these data were statistically insignificant.

## 2. Methods

### 2.1. Animals and Diet Conditions

Nine-week-old male and female C57BL6/J mice were fed a sterile HFD (HFD-60, KBT Oriental KK, Japan) or HFD-60 supplemented with BoE (see Table 1 and Supplementary Table 1, purchased from Ace Trading Co., Ltd., Japan). Mice fed the HFD-BoE diet were divided into groups that were fed 50 mg/kg or 500 mg/kg of HFD60. Body weight and dietary consumption were measured each week for 3 months (weeks 0–11, 12 measurements). The Animal Ethics Committee of the Oita University approved the protocol (G010002) for using mice (justified numbers, daily care, treatment, and euthanasia procedures).

### 2.2. Abdominal Computed Tomography (CT)

Body fat (both visceral and subcutaneous) of each mouse was quantitated using an RmCT2 3D micro X-ray CT scanner (Rigaku KK, Japan). On weeks 0, 4, 8, and 11, the amounts of visceral and subcutaneous adipose tissues between the fourth (L4) and fifth lumbar (L5) vertebral regions were determined after mice were anesthetised with isoflurane. The level of the L5 vertebra was defined as the line between the two highest points on the iliac crest.

### 2.3. Biochemical Analysis of Sera

A DRI-CHEM 4000 (Fuji Film KK, Japan) was used to quantify the biochemical markers in sera as follows: liver disorders: aspartate transaminase, alanine transaminase, and lactate dehydrogenase; heart function: creatine phosphokinase; and hyperlipidaemia: total cholesterol and triglycerides (TGs).

### 2.4. Quantitation of Cytokines/Chemokines

Aliquots of serum (12.5 *µ*l) from each mouse were collected (*n* = 8 per group). Quantitation of 23 cytokines and chemokines [IL-1*α*, IL-1*β*, IL-2, IL-3, IL-4, IL-5, IL-6, IL-9, IL-10, L-12 (p40), IL-12 (p70), IL-13, IL-17, eotaxin, G-CSF, GM-CSF, IFN-*γ*, KC, CP-1 (MCAF), MIP-1*α*, MIP-1*β*, RANTES, and TNF-*α*] was performed using a multiplex enzyme-linked immunosorbent assay (ELISA) system (Bio-Plex23, BioRad) and Bio-Plex Manager Software 6.1 (Bio-Rad) with a five-parameter curve-fitting algorithm for standard curve calculations as previously described [12]. The average value of each cytokine or chemokine was determined by excluding the highest and lowest outliers (*n* = 6 per group).

### 2.5. RNA Isolation and Real Time-Quantitative Polymerase Chain Reaction (RT-qPCR)

Total RNAs were purified from the lower abdominal adipose tissues using the RNeasy Mini Kit (QIAGEN KK, Japan). RNA quantity and purity were evaluated using a NanoDrop 2000 (Thermo Fisher Scientific K.K.). The TaqMan quantitative RT-qPCR assay with universal probe (UPL#80 for GAPDH and UPL#34 for UCP-1, Roche KK, Japan) was performed to validate a subset of genes (Table 2). The cDNAs were synthesized using purified RNAs by ReverTra Ace (TOYOBO KK, Japan) and random oligonucleotide (hexamers) primers. The products of the RT-qPCR reactions were quantitated using Light-Cycler R 480 System (Roche KK, Japan). Each reaction was performed in triplicate using* GAPDH* primers in the same reaction plate [13].

### 2.6. Statistical Analysis

All data are presented as means ± SD (*n* = 8), and homogeneity of variance of data in each group was analyzed using Bartlett's tests. After evaluating the homogeneity of variance in each data, the data among the male and female three groups were analyzed using one-way analysis of variance (ANOVA) and nonparametric test (Kruskal–Wallis test). Statistical differences between the HD controls and BoE-supplemented groups were analyzed using the Student *t*-test to confirm the significant differences in each of measurable criteria. All analyses were performed using a free software EZR version 1.35 package. A difference of *P* < 0.05 was considered statistically significant [14].

## 3. Results

### 3.1. Summary of Experimental Procedures

**Figure 1:**
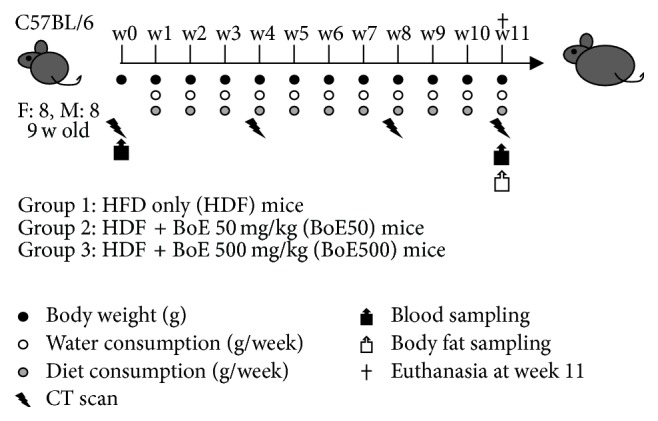
*Schematic representation of the experiments*. Each experimental group comprised eight 9-week-old C57BL/6 female and male mice. The control group was fed a high-fat diet (HFD-60) (Table 1) and was designated HFD. Two doses of condensed fermentative extracts of bonito (BoE) were used as supplements. BoE-50 was defined as the normal dose equivalent to that for humans (50 mg/kg), and BoE-500 was designated the high dose to evaluate anticipated adverse effects. Each biological activity was quantitated at the indicated intervals.

To evaluate the effects of BoE on obesity, we induced characteristic symptoms of obesity by feeding mice a HFD. HFD60 consists of 60% caloric content of fat and efficiently induces a 40% increase in body weight and a 100% increase in blood sugar levels according to the manufacturer's information (http://www.oyc.co.jp/en/). We used the HFD60 mice (HFD mice) as the control group (eight male and female mice in each group), and the test groups were fed HFD60 supplemented with 50 mg BoE/kg HFD (BoE50 mice) or 500 mg BoE/kg HFD (BoE500 mice), respectively. The mice were maintained on these diets for 11 weeks, and the biological effects of BoE were evaluated according to the schedule shown in Figure 1.

### 3.2. BoE Suppressed the HFD-Induced Increase in Body Weight

**Figure 2:**
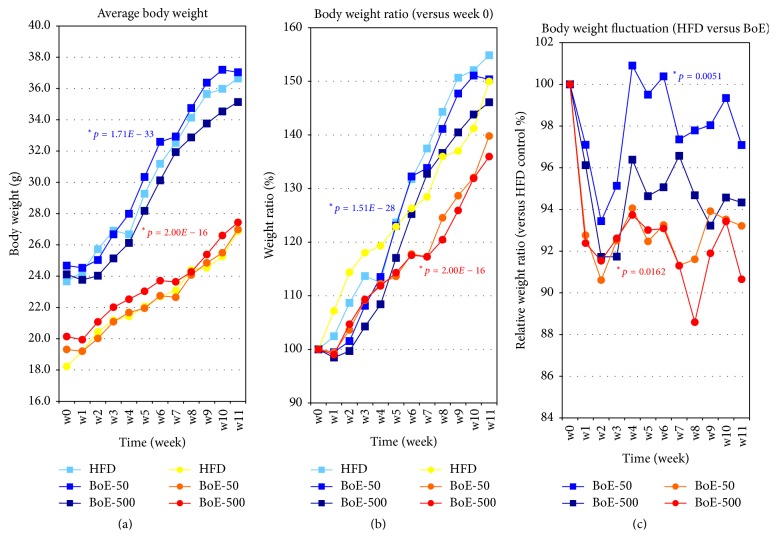
*Effects of BoE supplementation on body weight of mice fed a high-fat diet*. (a) Average weight of each experimental group from weeks 0 to 11. Each colour represents the male control (light blue), BoE-50 (blue), BoE-500 (navy), female control (yellow), BoE-50 (orange), and BoE-500 (red). Values were calculated from individual measurements of body weight. (b) Body-weight ratios. The average body weights of the six experimental groups at week 0 were defined as 100%, and the relative ratios from weeks 1 to 11 are shown. (c) The fluctuation of body-weight ratios between high-fat diet (HFD) control and BoE test group. The values of the control groups = 100% throughout the experiments, and the relative values of BoE-50 and BoE-500 for male and females are shown. The statistical significance of each criterion was evaluated by ANOVA and indicated as (^*∗*^*P* values).

The average weights and relative increases of the ratios of body weights of male and female test groups are shown in Figure 2. The data used to generate the plots are presented in Supplementary Table 2AB. Compared with controls, the average body weights (Figure 2(a)) and relative increases in the ratios of body weights (Figure 2(b)) of the BoE-supplemented groups were suppressed at most time points throughout the experiment. The relative ratios of the body weights of the controls versus BoE-supplemented groups are shown in Figure 2(c). The largest differences between controls and BoE-supplemented mice were 8.3% for males (BoE-500, week 2) and 11.4% for females (BoE-500, week 8) (see also Table 3 and Supplementary Table 2C–I).

### 3.3. Female Mice Consumed the Highest Quantities of HFD-BoE

**Figure 3:**
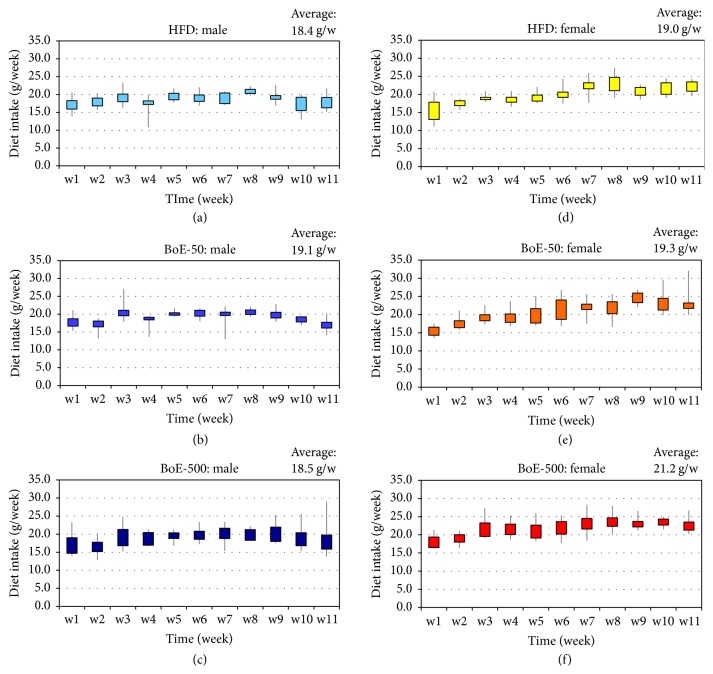
*Weekly consumption of the high-fat diet with or without BoE*. The average amount of diet consumed each week was calculated for each group. Male high-fat diet (HFD) group (a), BoE-50 group (b), and BoE-500 group (c). The corresponding female groups are designated (d–f). The average amount of diet consumed per week for six experimental groups is indicated in the top upper-right of each column.

The amount of dietary intake of each mouse is shown in Figure 3. The average quantities consumed by the male HFD control, BoE-50, and BoE-500-supplemented groups were 18.4, 19.1, and 18.5 g per week, respectively, and 19.0, 19.3, and 21.2 g per week for females. The female BoE-500 group consumed a significantly higher amount than the other groups [BoE-500 versus HFD (*P* = 5.74 × 10^−6^) or BoE-50 (*P* = 0.0234)]. In contrast, there was no significant difference among the groups of male mice (Table 4). These results suggest possible antiobesity effects on females because a statistically significant increase in dietary intake did not contribute to an increase in body weight of the female BoE-500 group.

### 3.4. BoE Suppressed the Accumulation of Visceral Adipose Tissue Induced by HFD

**Figure 4:**
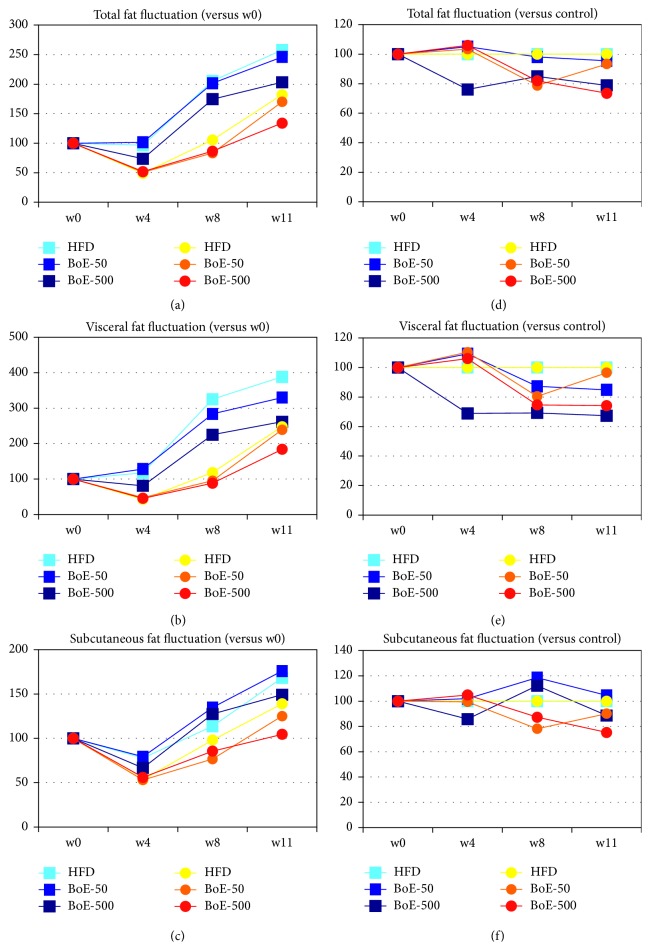
*Induction of body fat reduction by* BoE* supplementation*. Increasing ratios of body fat. Average amounts of total fat (a), visceral fat (b), and subcutaneous fat (c) of the six experimental groups at week 0 = 100%, and the relative ratios at weeks 1, 4, 8, and 11 are shown. The fluctuation of body fat ratios of total fat (d), visceral fat (e), and subcutaneous fat (f) between high-fat diet control and BoE test groups. The values of the control groups = 100% throughout the experiments and the relative values of BoE-50 and BoE-500 for male and females are shown.

Although there was a significant difference at only a few sampling times, each BoE-supplemented group exhibited a reduction in body weight (Figure 2). Because BoE contains multiple ingredients with antiobesity effects, including DHA, EPA, and anserine (Supplementary Table 1), we therefore determined whether the HFD-induced accumulation of body fat was inhibited by BoE (Figure 4). Independent of sex, the HFD control groups exhibited the highest accumulation of total and visceral body fat (Figures 4(a) and 4(b)) in a time-dependent manner, although the accumulation of subcutaneous fat was not suppressed in the BoE-50 group of male mice (Figure 4(c)). BoE-500 supplementation reduced the accumulation of total and visceral fat (Figures 4(d) and 4(e)); however, the difference was statistically significant only in total fat (week 8) for the BoE-500 female and in visceral fat (week 11) for the BoE-500 male mice groups (Supplementary Table 3). There were no significant differences associated with the subcutaneous fat (Supplementary Table 3).

### 3.5. BoE Reduced the Serum Levels of TGs, Total Cholesterol, and Inflammatory Cytokines

Because BoE supplementation reduced weight gain and the accumulation of visceral fat, we further assessed the effects of BoE by quantitating the serum components of each mouse. The differences of the values of six components (AST, ALT, LDH, CPK, TCHO, and TG) associated with liver and heart function and obesity were not significantly different between the HFD and HFD-BoE groups. However the accumulations of TCHO and TG were significantly higher in male mice than female which suggested a sex-related obesity tendency with HFD and this tendency could not be altered even with BoE supplementation (Table 5 and Supplementary Table 4). Additionally, a long-term intake of BoE did not affect liver or heart function, although BoE-500, which is ten times higher than the equivalent human dose, contains relatively high amounts of sodium chloride and arsenic.

Obesity caused by excess fat and sugars stimulates the secretion of multiple cytokines called adipocytokines [15] that are closely associated with cardiovascular disorders such as arteriosclerosis, hypertension, and cerebrovascular disease [16]. To evaluate the potential protective or anti-inflammatory effects of BoE on blood circulation system of HFD mice, we determined the levels of 23 cytokines and chemokines in mice sera of each group by multiplex enzyme-linked immunosorbent assay (ELISA) system. As we expected, long-term HFD intake (without BoE supplementation) induced significant levels of cytokines (see Supplementary Table 5, IL-2, IL-3, IL5, IL-6, IL-12(p49), IL-13, IL-17, eotaxin, G-CSF, GM-CSF, IFN-*γ*, KC, MCP-1, MIP-1*β*, RANTES, and THF*α* in male and IL-1*α*, IL-1*β*, IL-2, IL-3, IL-4, IL-5, IL-6, IL-12(p49), IL-12(p70), IL-13, IL-17, eotaxin, G-CSF, GM-CSF, IFN-*γ*, MCP-1, MIP-1*α*, MIP-1*β*, RANTES, and TNF*α* in females. *P* values in red characters indicate significant differences between week 0 and week 11). To the multiple induction of inflammatory cytokines, BoE-500 supplementation had suppressive effects on the levels IL-5, IL-6, and IL-13, while BoE-50 had an effect on G-CSF level. These were observed only in female mice (Table 6). Since BoE-50 is equivalent to the recommended dose for humans, G-CSF was the only molecule to decrease with BoE supplementation. However, levels of IL-5, IL-6, and IL-13 went down in a dose-dependent manner and it could be interpreted that BoE supplementation could ameliorate the obesity related inflammatory features in the circulation system.

### 3.6. BoE-Induced UCP-1 Expression in Mouse Adipocytes

Uncoupling proteins (UCPs) localize to the inner surface of the mitochondria to generate heat through nonshivering thermogenesis. UCP-1 was first discovered in the mitochondria of brown adipose tissues that impart this tissue with enormous heat-generating capacity [11]. More recently, a thermogenic tissue called beige adipose tissue was identified [17, 18]. Both tissues generate heat upon receiving extracellular stimulatory signals such as cold stress.

**Figure 5:**
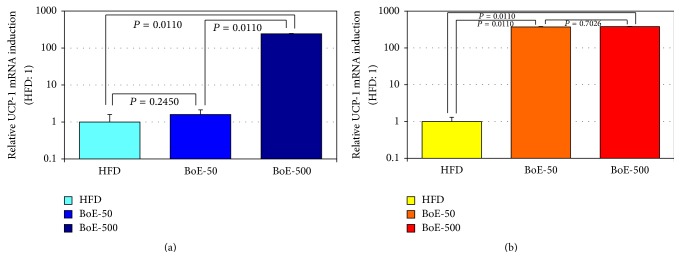
*Induction of UCP-1 mRNA in the abdominal adipose tissues by BoE supplementation*. Adipose tissues from the genital area were collected for RNA preparation at the end of the experiment. The expression of* UCP-1* mRNA in each adipose tissue of male (a) and female (b) mice was quantitated as described in experimental methods. The statistical significance of each criterion was evaluated by nonparametric test (Kruskal–Wallis test).

We next assessed the levels of* UCP-1* mRNA in visceral adipose tissues (Figure 5).* UCP-1* levels in visceral adipose tissues were significantly increased in all but one group (male HFD- BoE-50) by the BoE diets. The induction levels of* UCP-1* mRNA by BoE supplementation were as follows: 1.59 and 241.89 for male and 372.16 and 379.38 for female BoE-50 and BoE-500 groups, respectively (Figure 5 and Table 7).

## 4. Discussion

BoE consists of a mixture of fermented, dried bonito meat, and condensed boiled bonito soup, which has served for centuries as a seasoning or nutritional supplement in the Southern District of Kagoshima prefecture, Kyushu Island, Japan [1]. One gram of BoE contains 638 mg of amino acids, 5.3 mg of DHA, 0.8 mg of EPA, 1.12 mg of niacin (cardiovascular protection) [19], 6.3 mg of anserine (radical scavenger), and 14.4 mg of taurine (liver function and anti-inflammation). Although it consists of multiple ingredients that maintain and could improve health, BoE also contains relatively high amounts of sodium chloride (300 mg/g) and arsenic (23 ppm) that might cause adverse effects on health. We therefore assessed BoE's health-promoting activity with a focus on its antiobesity effects, which is defined by the reduction of visceral fat and total cholesterol in sera. We assessed BoE's potential anti-inflammatory activity using a multiplex ELISA system that detects 23 cytokine/chemokines associated with inflammation and assessed BoE's safety by measuring biochemical markers of liver and heart function.

We measured body weight, dietary, and water consumption each week to establish baseline values. Monthly CT scans were collected, blood sampling was conducted twice at the start and end of the study, and visceral fat tissues were analyzed at the end of the study. BoE is commercially available, and the supplier recommends 300 mg/day (42 mg/kg/week for men and 30 mg/kg/week for women). We decided to use an established experimental mouse model of obesity [20] using a dose of 50 mg per kg body weight per week (mg/Kg/week: equivalent to the human dose) and 500 mg/Kg/week of BoE to evaluate its antiobesity and anti-inflammatory effects and its safety, especially with higher dose groups.

Here, we show that BoE significantly reduced the weight gain induced by a HFD. Specifically, the BoE-50 (week 2) and BoE-500 (weeks 2 and 3) groups of male mice achieved a statistically significant reduction in body weight. The corresponding groups of female mice achieved larger reductions in weight (BoE-50, –9.4%, week 2; BoE-500, –11.4%, week 8) than male mice. While one-by-one comparison between HFD and BoE-supplemented group did not show statistically significant differences because of the wide variations in the individual weight of the HFD groups (Supplementary Table 2), additional ANOVA and nonparametric test (Kruskal–Wallis test) analysis [14] clearly demonstrated BoE's weight reducing effects in both sexes (Figure 2). Because of its extremely high-caloric input, marked induction of adipose tissue growth was observed particularly in the visceral area of all individual mouse even supplemented with BoE (Figure 4 and Supplementary Table 3); however each BoE-supplemented group achieved a decrease in the amount of visceral fat throughout the study (Figures 4(b) and 4(e) and Supplementary Table 3). Statistical significance could not be achieved (except for of BoE-500 male mice) because of high individual variations in this experiment.

Biochemical analysis and multiplex ELISA quantitation for sera revealed accumulation of TCHO (Table 5), strong induction of inflammatory cytokines by HFD intake (Table 6 and Supplementary Table 5), and BoE supplementation significantly reduced levels of IL-5, IL-6, IL-13, and G-CSF (Table 6). IL-5 activates eosinophils and plasmacytes to facilitate the immune response, and its overexpression causes obese individuals to become severely asthmatic [21]. IL-6 activates the immune response through inducing the maturation of B cells; induces the expression of cell adhesion molecules, which contribute to the ability of immune cells to infiltrate tissues; and suppresses the activation of regulatory T cells. Overexpression of IL-6 therefore induces multiple inflammatory symptoms, chronic fatigue, insomnia, and obesity [22, 23]. IL-13 exacerbates IL-5-triggered asthma [24], and overexpression of IL-13 in the intestinal tract induces inflammatory bowel disease [25] and causes lesions of the endothelium, which eventually lead to arteriosclerosis and other cardiovascular dysfunctions [26]. G-CSF induces the growth of granulocytes and contributes to the regeneration of endothelial cells [27] and the highly increased ratio of G-CSF levels among the 23 ELISA assay could explain its repairing role on the endothelial lesions caused by HFD intake. BoE supplementation reduced levels of multiple inflammatory cytokines in the circulation system; therefore, the requirement for G-CSF activity may be diminished in the BoE-supplemented mice.

We were surprised with unexpectedly high* UCP-1 *mRNA expression in visceral tissues of BoE-supplemented mice (Figure 5 and Table 7). The recent identification of beige adipose tissue (BeAT) [17] changed the concept of dualism for fat-accumulating white adipose (WAT) and fat-burning brown adipose tissue (BeAT). BAT normally behaves in a similar manner to WAT, but once it is subjected to a fat-burning signal such as cold stimulation, BeAT rapidly differentiates into BAT-like tissue. Although we do not know the molecular machinery that mediates BoE-induced* UCP-1* expression at this moment, Ochiai and his colleagues reported that peptides from the delipidized fermented bonito extract (without EPA/DHA) still possessed ameliorating function for type 2 DM mice and unidentified peptides or other ingredients (Supplementary Table 1) might interact with cell surface receptors, such as thyroid-stimulating hormone receptor [28] or glucagon-like peptide-1 receptor [29] to trigger the fat-burning thermogenesis.

Evidence from epidemiological surveys clearly indicates a relationship between obesity and multiple chronic diseases, including cancer [30–32], cardiovascular disorders [33–35], and neurological and behavioural disorders [36, 37]. Bonito extracts ameliorated the scores of mental fatigue and sleep disruption [38]. All this accumulating evidence may support the antiobesity and anti-inflammatory effects of BoE on our experimental animal model although the responsible molecules for these activities are still elusive. Further investigation for identification should be continued.

## Supplementary Material

Table 1: Composition and estimated calories of BoE.
Table 2: Body weights of six high fat diet feeding mice groups supplemented with or without BoE.
Table 3: CT-scan quantitation of A, total; B, visceral and C, subcutaneous fat amount between the fourth and the fifth lumbar vertebra region.
Table 4: Quantitation of six biomarkers related with liver and heart functions and obesity.
Table 5: Multiplex quantitation of inflammation related cytokine, chemokine and growth factors secreted in sera.

## Figures and Tables

**Table 1:** Composition and estimated calories of HFD-60 and its BoE-supplemented diet.

Control HFD		BoE-50	BoE-500
Milk casein	256.0	HFD^*∗*^	HFD^*∗*^
L-Cystine	3.6		
Lard	330.0	**BoE**	**BoE**
Soybean oil	20.0	**42**	**420**
*α*-Cornstarch	160.0		
Powdered cellulose	66.1		
Malt dextrin	60.0		
Sucrose	55.0		
AIN-93G-MX	35.0		
AIN-93-VM	10.0		
Choline bitartrate	2.5		
Calcium carbonate	1.8		

	g/kg diet	*mg/kg diet*	*mg/kg diet*

Protein	922		
Fat	3150		
Carbohydrate	990		
Fiber	0		
Total calorie	5062	HFD^*∗*^	HFD^*∗*^

kcal/kg diet;  ^*∗*^same composition as HFD-60.

**Table 2:** Primers and UPL probes used for RT-PCR.

	Left	Right	UPL probe number
GAPDH	tgtccgtcgtggatctgac	cctgcttcaccaccttcttg	#80
UCP-1	ggcctctacgactcagtcca	taagccggctgagatcttgt	#34

**Table 3:** Body weight fluctuation induced by BoE supplementation.

Sex	Group	w0	w1	w2	w3	w4	w5	w6	w7	w8	w9	w10	w11
M	BoE-50	0.00%	−2.90%	−6.56%	−4.86%	0.90%	−0.50%	0.38%	−2.64%	−2.21%	−1.96%	−0.66%	−2.91%
		ND	*P* = 0.2778	**P** = 0.0282	*P* = 0.0765	*P* = 0.8195	*P* = 0.8898	*P* = 0.9138	*P* = 0.5997	*P* = 0.6366	*P* = 0.6312	*P* = 0.8850	*P* = 0.4551
	BoE-500	0.00%	−3.88%	−8.28%	−8.27%	−3.62%	−5.36%	−4.94%	−3.43%	−5.32%	−6.78%	−5.43%	−5.67%
		ND	*P* = 0.2811	**P** = 0.0042	**P** = 0.0147	*P* = 0.3807	*P* = 0.1841	*P* = 0.2505	*P* = 0.4324	*P* = 0.2392	*P* = 0.1246	*P* = 0.2633	*P* = 0.1503

F	BoE-50	0.00%	−7.24%	−9.39%	−7.49%	−5.94%	−7.53%	−6.75%	−8.69%	−8.39%	−6.09%	−6.48%	−6.79%
		ND	*P* = 0.2940	*P* = 0.2259	*P* = 0.2286	*P* = 0.3138	*P* = 0.2346	*P* = 0.2622	*P* = 0.1583	*P* = 0.2505	*P* = 0.4200	*P* = 0.4198	*P* = 0.4203
	BoE-500	0.00%	−7.62%	−8.46%	−7.39%	−6.27%	−6.99%	−6.91%	−8.70%	−11.41%	−8.10%	−6.58%	−9.35%
		ND	*P* = 0.2662	*P* = 0.2704	*P* = 0.2308	*P* = 0.2873	*P* = 0.2553	*P* = 0.2416	*P* = 0.1426	*P* = 0.0961	*P* = 0.2716	*P* = 0.3973	*P* = 0.2495

**Table 4:** Statistical significance between diet consumption and BoE supplementation.

Sex	Group	Diet consumption	*P* value	*P* value
		(average g/week)	(versus HFD)	(BoE-50 versus BoE-500)
M	HFD	18.4	ND	ND
	BoE-50	19.1	*P* = 0.1189	
	BoE-500	18.5	*P* = 0.2475	*P* = 0.8610

F	HFD	19.0	ND	ND
	BoE-50	19.3	*P* = 0.0723	
	BoE-500	21.2	**P** = 5.74**E** − 06	**P** = 0.0234

**Table 5:** Biochemical examination of six markers for liver and heart function or obesity.

Subject	Sex	Group	Average	Std. dev.	Versus HFD	M versus F
					*P* value	*P* value
AST	M	HFD	101.17	45.19		*P* = 0.9530
		BoE-50	108.17	38.97	*P* = 0.7910	*P* = 0.1106
		BoE-500	103.67	44.19	*P* = 0.9283	*P* = 0.4092
	F	HFD	102.67	35.27		
		BoE-50	74.83	18.80	*P* = 0.1437	
		BoE-500	82.67	34.97	*P* = 0.3694	

ALT	M	HFD	30.83	16.24		*P* = 0.2757
		BoE-50	29.83	12.86	*P* = 0.9134	*P* = 0.4884
		BoE-500	22.50	4.55	*P* = 0.2803	*P* = 0.7455
	F	HFD	22.50	3.83		
		BoE-50	24.67	10.54	*P* = 0.6528	
		BoE-500	21.00	9.82	*P* = 0.7393	

LDH	M	HFD	413.17	137.01		*P* = 0.0995
		BoE-50	473.00	189.66	*P* = 0.5580	*P* = 0.0527
		BoE-500	378.17	83.03	*P* = 0.6280	*P* = 0.4564
	F	HFD	298.00	47.12		
		BoE-50	273.50	72.19	*P* = 0.5142	
		BoE-500	460.67	239.93	*P* = 0.1641	

CPK	M	HFD	548.17	516.19		*P* = 0.4599
		BoE-50	408.33	222.12	*P* = 0.5645	*P* = 0.0715
		BoE-500	401.83	299.19	*P* = 0.5876	*P* = 0.6351
	F	HFD	369.67	200.76		
		BoE-50	190.17	105.17	*P* = 0.1014	
		BoE-500	325.00	201.21	*P* = 0.7212	

TCHO	M	HFD	169.33	49.46		**P** = 0.0320
		BoE-50	155.50	44.72	*P* = 0.6399	*P* = 0.0582
		BoE-500	128.50	43.87	*P* = 0.1819	*P* = 0.4216
	F	HFD	109.00	8.20		
		BoE-50	109.17	12.40	*P* = 0.9793	
		BoE-500	108.83	31.54	*P* = 0.9905	

TG	M	HFD	94.33	15.20		**P** = 7.08**E** − 04
		BoE-50	106.50	20.73	*P* = 0.2886	**P** = 0.0039
		BoE-500	108.50	29.41	*P* = 0.3288	**P** = 0.0080
	F	HFD	54.50	11.55		
		BoE-50	64.33	15.72	*P* = 0.2602	
		BoE-500	58.00	19.08	*P* = 0.7159	

**(a):** 

IL-5	Average	Relative	Standard	*P* value	*P* value
	value	value	deviation	w0 versus w11	HFD versus BoEs
M					
HFD-start	51.2	1	13.4	*P* = 0.0421	
HFD-end	95.0	1.855	31.7		
BoE50-start	53.8	1	4.7	*P* = 0.3271	*P* = 0.1876
BoE50-end	71.2	1.323	38.0		
BoE500-start	50.0	1	13.4	*P* = 0.2002	*P* = 0.3109
BoE500-end	66.9	1.338	28.4		
F					
HFD-start	69.5	1	14.5	*P* = 0.0010	
HFD-end	134.6	1.937	10.2		
BoE50-start	62.3	1	14.3	*P* = 0.0014	*P* = 0.6964
BoE50-end	115.0	1.846	10.7		
BoE500-start	65.1	1	9.5	*P* = 0.3074	**P** = 0.0118
BoE500-end	78.4	1.204	24.4		

**(b):** 

IL-6	Average	Relative	Standard	*P* value	*P* value
	value	value	deviation	w0 versus w11	HFD versus BoEs
M					
HFD-start	20.8	1	5.4	*P* = 0.0247	
HFD-end	39.6	1.904	9.4		
BoE50-start	22.9	1	2.4	*P* = 0.0789	*P* = 0.1891
BoE50-end	33.4	1.459	11.9		
BoE500-start	21.6	1	3.2	*P* = 0.1137	*P* = 0.3341
BoE500-end	33.5	1.551	14.5		
F					
HFD-start	26.1	1	4.8	*P* = 0.0010	
HFD-end	49.0	1.877	6.9		
BoE50-start	25.3	1	8.4	*P* = 0.0174	*P* = 0.5091
BoE50-end	49.0	1.937	5.4		
BoE500-start	25.6	1	4.0	*P* = 0.2237	**P** = 0.0120
BoE500-end	30.8	1.203	9.3		

**(c):** 

G-CSF	Average	Relative	Standard	*P* value	*P* value
	value	value	deviation	w0 versus w11	HFD versus BoEs
M					
HFD-start	71.9	1	17.0	*P* = 0.0140	
HFD-end	218.3	3.036	81.4		
BoE50-start	78.7	1	13.2	*P* = 0.0026	*P* = 0.1868
BoE50-end	177.7	2.258	64.9		
BoE500-start	81.7	1	11.0	*P* = 0.0540	*P* = 0.1789
BoE500-end	164.7	2.016	72.8		
F					
HFD-start	103.6	1	25.1	*P* = 4.82*E* − 04	
HFD-end	221.6	2.139	42.2		
BoE50-start	117.4	1	24.2	*P* = 0.0023	**P** = 0.0250
BoE50-end	189.8	1.617	18.9		
BoE500-start	106.1	1	19.0	*P* = 0.1256	*P* = 0.0561
BoE500-end	162.8	1.534	49.3		

**(d):** 

IL-13	Average	Relative	Standard	*P* value	*P* value
	value	value	deviation	w0 versus w11	HFD versus BoEs
M					
HFD-start	724.3	1	198.4	*P* = 0.0429	
HFD-end	1248.3	1.723	467.6		
BoE50-start	790.6	1	139.7	*P* = 0.2284	*P* = 0.2425
BoE50-end	1053.4	1.332	493.4		
BoE500-start	752.8	1	63.2	*P* = 0.1103	*P* = 0.4582
BoE500-end	1099.6	1.461	420.0		
F					
HFD-start	889.7	1	237.4	*P* = 0.0037	
HFD-end	1852.5	2.082	315.1		
BoE50-start	950.6	1	266.1	*P* = 0.0107	*P* = 0.6692
BoE50-end	1786.2	1.879	210.0		
BoE500-start	977.9	1	168.6	*P* = 0.1396	**P** = 0.0243
BoE500-end	1275.9	1.305	462.6		

**Table 7:** Statistical significance on BoE-induced UCP-1 expression in the visceral tissues.

Group	Sex	Average	SD	HFD versus BoE	Male versus female
		Versus HFD	Relative SD	*P* value	*P* value
HFD	M	7.40*E* − 05	4.32*E* − 05		*P* = 0.3609
		1.00	0.58		
BoE-50		1.18*E* − 04	6.24*E* − 05	*P* = 0.2177	*P* = 0.0054
		1.59	0.53		
BoE-500		1.79*E* − 02	8.86*E* − 04	**P** = 0.0104	*P* = 0.0157
		241.89	4.95		

HFD	F	9.70*E* − 05	2.95*E* − 05		
		1.00	0.3		
BoE-50		3.61*E* − 02	3.92*E* − 03	**P** = 0.0054	
		372.16	10.86		
BoE-500		3.68*E* − 02	2.75*E* − 03	**P** = 2.19**E** − 06	
		379.38	7.47		

## Abbreviations

BoE: Condensed fermentative extracts of bonito
anserine: *β*-Alanyl-N-methylhistidine
DHA: Docosahexaenoic acid
EPA: Eicosapentaenoic acid
HFD: High-fat diet
AST: Aspartate aminotransferase
ALT: Alanine transaminase
LDH: Lactate dehydrogenase
CPK: Creatine phosphokinase
TCHO: Total cholesterol
TG: Triglycerides
IL: Interleukin
G-CSF: Granulocyte-colony stimulating factor
CT scan: Computed tomography scan
RT-qPCR: Real time-quantitative polymerase chain reaction
UCP-1: Uncoupling protein 1
GAPDH: Glyceraldehyde 3-phosphate dehydrogenase.
