# Supplementary material for: Antiobesity and Anti-Inflammatory Effects of Orally Administered Bonito Extracts on Mice Fed a High-Fat Diet
Source: Evid Based Complement Alternat Med. 2017 Oct 24;2017:9187167. doi: 10.1155/2017/9187167 (PMC5674501; doi:10.1155/2017/9187167)
Supplement: Supplementary file 1 — Table 1: Composition and estimated calories of BoE. Table 2: Body weights of six high fat diet feeding mice groups supplemented with or without BoE. Table 3: CT-scan quantitation of A, total; B, visceral and C, subcutaneous fat amount between the fourth and the fifth lumbar vertebra region. Table 4: Quantitation of six biomarkers related with liver and heart functions and obesity. Table 5: Multiplex quantitation of inflammation related cytokine, chemokine and growth factors secreted in sera. [file 9187167.f1.zip › 170829 Supplements information.docx]

**Supplements information.**

**Table. 1. Composition and estimated calories of BoE.** The composition of all ingredients were quantitated by Pharmatech Co. Ltd. Tokyo. Japan.

**Table 2. Body weights of six high fat diet feeding mice groups supplemented with or without BoE. A)** Body weight of individual mouse was monitored every week. **B)** Relative body weight fluctuation was calculated from Table 2A. **C)** Average weights of six groups were calculated from Table 2A. **D)** Relative average body weight fluctuation was calculated from Table 2C. **E)** A permutation of Table 2D values to percentage. **F)** Standard deviation for each average value. **G)** Relative body weight ratios of BoE50 or BoE500 groups to HFD control at each time point. **H)** Relative body weight ratio fluctuation of BoE50 or BoE500 groups to HFD control at each time point. Yellow background represents biggest gaps between control and BoE group. **I)** Significance of Relative body weight ratio fluctuation of BoE50 or BoE500 groups to HFD control at each time point. Yellow background represents statistical significance (p<0.05).

**Table 3. CT-scan quantitation of A, total; B, visceral and C, subcutaneous fat amount between the fourth and the fifth lumbar vertebra region. Sheet 1 (CAT Wk0 vs Wk4)** represents each calculation of **A)** total; **B)** visceral and **C)** subcutaneous fat amount ration between week 0 and week 4. Likewise, **Sheet 2 (CAT Wk0 vs Wk8)** represents ratio between week 0 and week 8, **Sheet 3 (CAT Wk0 vs Wk11)** represents ratio between week 0 and week 11.

**Table 4. Quantitation of six biomarkers related with liver and heart functions and obesity.** As described in materials and methods, six biochemical markers (AST, ALT, CPK, TCHO, TG and LDH) in sera of each mice quantitated by Fuji DryChem System. **Panel A** represents individual data and **panel B** represents average values, standard deviation and p-values of panel A. Yellow background represents statistical significance (p<0.05).

**Table 5. Multiplex quantitation of inflammation related cytokine, chemokine and growth factors secreted in sera.** The amount of each protein in sera was quantitated by multiplex ELISA system (Bio-Plex23, BioRad) and calculated its significance by Bio-Plex Manager software ver. 6.1 (BioRad). Difference between two time points, week 0 and week 11 of the average values of six mice groups were displayed in each panel. Yellow background represents statistical significance (p<0.05) between week 0 and week11 or among HFD, BoE50 and BoE500 groups.
